# Study on the SHP2-Mediated Mechanism of Promoting Spermatogenesis Induced by Active Compounds of Eucommiae Folium in Mice

**DOI:** 10.3389/fphar.2022.851930

**Published:** 2022-03-22

**Authors:** Hailong Mu, Shuangshi Liu, Shiyang Tian, Beibei Chen, Zengyuan Liu, Yunpeng Fan, Yingqiu Liu, Wuren Ma, Weimin Zhang, Mingzhe Fu, Xiaoping Song

**Affiliations:** College of Veterinary Medicine, Northwest A&F University, Yangling, China

**Keywords:** Eucommiae Folium, spermatogenesis, CGA, SHP2, spermatogonial stem cell, leydig cell

## Abstract

Spermatogenesis directly determines the reproductive capacity of male animals. With the development of society, the increasing pressure on people’s lives and changes in the living environment, male fertility is declining. The leaf of *Eucommia ulmoides* Oliv. (Eucommiae Folium, EF) was recorded in the 2020 Chinese Pharmacopoeia and was used in traditional Chinese medicine as a tonic. In recent years, EF has been reported to improve spermatogenesis, but the mechanisms of EF remain was poorly characterized. In this study, the effect of EF ethanol extract (EFEE) on spermatogenesis was tested in mice. Chemical components related to spermatogenesis in EF were predicted by network pharmacology. The biological activity of the predicted chemical components was measured by the proliferation of C18-4 spermatogonial stem cells (SSCs) and the testosterone secretion of TM3 leydig cells. The biological activity of chlorogenic acid (CGA), the active compound in EF, was tested *in vivo.* The cell cycle was analysed by flow cytometry. Testosterone secretion was detected by ELISA. RNA interference (RNAi) was used to detect the effect of key genes on cell biological activity. Western blotting, qRT–PCR and immunofluorescence staining were used to analyse the molecular mechanism of related biological activities. The results showed that EFEE and CGA could improve spermatogenesis in mice. Furthermore, the main mechanism was that CGA promoted SSC proliferation, self-renewal and Leydig cell testosterone secretion by promoting the expression of SHP2 and activating the downstream signaling pathways involved in these biological processes. This study provided strong evidence for elucidating the mechanism by which EF promotes the spermatogenesis in mice and a new theoretical basis for dealing with the decrease in male reproductive capacity.

## Introduction

Spermatogenesis is a process from SSC to mature sperm through mitosis and meiosis in the seminiferous tubules (Haseeb et al., 2019; Yang and Yang, 2020). The process involves autocrine, paracrine, and other hormonal stimuli and nutrients that are supportive of the germ cells development (Ehmcke and Schlatt, 2006; Dube and Cyr, 2012). The maintenance of spermatogenesis relies on the production of testosterone and follicle stimulating hormone (FSH) (Ramaswamy and Weinbauer, 2014). Testosterone is the cornerstone of spermatogenesis, secondary sexual characteristics and functions (Huhtaniemi, 2018). FSH enhances testosterone action by maintaining the supporting function of Sertoli cells on spermatogenesis (Mclachlan et al., 2002). Testosterone is considered the master switch of spermatogenesis, FSH is known to contribute to the quality and quantity of the sperm (Oduwole and Huhtaniemi, 2014).

In leydig cell, luteinizing hormone (LH) binds luteinizing hormone recepter (LHR) and stimulates cAMP pathway. The cAMP pathway, through protein kinase A (PKA), is essential for regulating the expression of steroidogenic acute regulatory protein (StAR) which acts at the mitochondria to trigger cholesterol movement across the membranes (Papadopoulos and Miller, 2012; Clark, 2016). After cholesterol is transferred from the outer to the inner mitochondrial membrane, it is converted to testosterone by steroidogenic enzymes: CYP11A1, 3β-HSD, CYP17A1 and 17β-HSD (Payne and Hales, 2004). In addition to the well-established regulation of testosterone synthesis by PKA, several regulators were identified. These include the signaling molecules PDGF and DHH; the kinases MAPK, PKG, CAMKI, and AMPK; and the transcription factors NUR77, MEF2, and GATA4 (Tremblay, 2015).

The SSC self-renewal, which encompasses cell division and cell survival, maintains the stem cell pool. Glial cell line-derived neurotrophic factor (GDNF) is a key factor for maintenance of SSC self-renewal. GDNF acts through different signaling pathways to induce target genes that promote SSC self-renewal, such as PI3K/AKT, SFK, and MAPK signaling pathways (Lee et al., 2007; Oatley et al., 2007; Takashima et al., 2015). The well studied GDNF-inducible self-renewal genes include *Etv5*, *Bcl6b*, *Lhx1*, *Pou3f1* and *Id4*. Moreover, there are many GDNF-independent and SSC-derived factors such as PLZF, FOXO1, GILZ and TAF4B that also contribute to regulate the self-renewal of SSC (Song and Wilkinson, 2014).

SHP2 is a non-receptor protein tyrosine phosphatase that is encoded by protein tyrosine phosphatase non-receptor type 11 gene (*Ptpn11*). A core component of receptor tyrosine kinases (RTKs), cytokines, and G protein-coupled receptor signal transduction, SHP2 shows ubiquitous expression and plays critical roles in cellular growth, survival, proliferation, and migration. In testis, SHP2 played a critical rule for the proliferation and self-renewal of SSC in the process of spermatogenesis (Hu et al., 2015). Meanwhile, SHP2 could support the steroidogenesis in leydig cells leading to testosterone production and maintain the blood testis barrier, which provided a stable environment for spermatogenesis (Puri and Walker, 2016).

Spermatogenesis directly determines the reproductive capacity of male animals. With the development of society, the increasing pressure on people’s lives and changes in the living environment, male fertility is declining (Agarwal et al., 2021). Traditional Chinese medicine (TCM) is commonly used to improve spermatogenesis in China, such as Morindae officinalis Radix (Chen and Wang, 2015), Eucommiae Folium (Fu et al., 2019) Cynomorii Herba (Yang et al., 2010) and Epimedii Folium (Park et al., 2017).

In the monotypic genus Eucommia, *Eucommia ulmoides* Oliv. is known as Dù-zhòng (Chinese:杜仲), Tuchong (in Japanese), and Chinese rubber tree (Cronquist, 1981). According to the Chinese Pharmacopoeia and the Shennong’s Herbal Classic of Materia Medica, the leaf and bark of this plant have the similar efficacy, as a famous botanical tonics, have been widely used for long time. They can be used alone or mixed with other herbs in the prescription of TCM to treat impotence, spermatorrhoea, prospermia, kidney deficiency pain *etc.* (He et al., 2014). Ethnopharmacological studies have shown that EFEE could improve the reproductive capacity and the testosterone levels in male rats (Fu et al., 2019). Du zhongkangcha (consist of Eucommiae Folium, Psoraleae Fructus and Lycii Fructus) significantly increased the sexual capacity in male rats (Zhang et al., 1999). Yougui Pill (consist of Radix Rehmanniae Praeparata, Cuscutae Semen, Lycii Fructus, Eucommiae Cortex *etc.*) could effectively improve the spermatogenic dysfunction in male patients (Qiu, 2020; Zhai et al., 2020). Through network pharmacological analysis, Yougui Pill was involved in the treatment of sexual dysfunction through regulating MAPK signaling pathway (Wang Y. et al., 2019).

EF mainly contains lignans, iridoids, phenylpropanoids and flavonoids. The chemical components include aucubin, eucommiol, pinoresinol, quercetin, rutin, CGA and kaempferol (Zhang et al., 2013). However, the mechanism of its specific compounds, which are involved in spermatogenesis, is not clear. Studies have shown that quercetin could promote the expression of the *Star* gene, associated with testosterone secretion in the testicular leydig tumour cell line MA-10 (Cormier et al., 2017). Rutin could improve stem cell proliferation by enhancing the phosphorylation of the PI3K/AKT/mTOR signaling pathway (Zhao et al., 2020). Kaempferol promoted stem cell proliferation through the Wnt signaling pathway (Nie et al., 2020). CGA could increase the number of sperm in rat testis (Park and Han, 2013). Moreover, CGA could effectively enhance the expression of the *Shp2* gene in stem cells and then promoted the expression of downstream cell proliferation-related genes (Zhou et al., 2016).

The pharmacological action of TCM is mainly related to its chemical components. However, very few studies have investigated the major components of EF associated with spermatogenesis. The uncertainty of chemical components related to spermatogenesis in EF limits its effectiveness in clinical applications. Therefore, the clarification of the main spermatogenic components of EF is of great significance for the further development.

In this study, the main active components related to spermatogenesis in EF and their signaling pathways were predicted by network pharmacology. The C18-4 cell proliferation assay and TM3 cell testosterone secretion assay were used to screen the active compounds in EF, and the active substances that could promote SSC proliferation and leydig cell testosterone secretion were selected. The regulation of spermatogenesis in mice was investigated by detecting the expression of key proteins in the signaling pathways related to SSC proliferation and self-renewal, Leydig cell testosterone secretion and cell proliferation. Our study will provide a theoretical basis for revealing the biochemical mechanism of spermatogenesis regulation by compounds in EF.

## Material and Methods

### Cell and Culture Materials

C18-4 cells were established by stably transfecting type A spermatogonia from 6-day-old mice with the large T antigen gene under the control of a ponasterone A-driven promoter (Hofmann et al., 2005; He et al., 2009), and they were cultured in DMEM/F12 supplemented with 10% FBS and maintained in a 5% CO_2_ atmosphere.

TM3 cells were purchased from the American Type Tissue Culture Collection (ATCC, Manassas, VA, United States) and maintained in a 5% CO_2_ atmosphere in DMEM/F12 (HyClone, United States) supplemented with 2.5% FBS (HyClone, United States), 5% HS (Solarbio Beijing, China) and 1% P/S solution (Solarbio Beijing, China).

### Cell Viability Assay

C18-4 cells were cultured in 96-well plates and treated with different concentrations of CGA (Cat. No. YZ-110753, HPLC ≥96.1%), quercetin (Cat. No. SQ8030, HPLC ≥98%), kaempferol (Cat. No. SK8030, HPLC ≥98%) and rutin (Cat. No. SR8250, HPLC ≥98%) (0–100 μM) (Solarbio Beijing, China). Thirty-six hours after treatment, 10 μl of WST-1 (Beyotime Biotechnology, Shanghai, China) solution per well was added and the plate was incubated for 1 h at 37°C. The absorbance of each well was measured at 450 nm by a microplate spectrophotometer. The cell viability was calculated using the following formula: Cell viability= (OD_treated group_−OD_blank group_)/(OD_control group_−OD_blank group_).

### Extraction and Isolation

EF (voucher specimen number: TCVM-15082501) was collected from Lueyang, Shaanxi Province (33°07′55″N, 105°42′31″E) and stored in the specimen room of Traditional Chinese Veterinary Medicine (TCVM) of the College of Veterinary Medicine. EF powder (160.0851 g) was refluxed for 30 min, at 97°C, with 1600 ml 50% ethanol solution (v/v). After repeated extraction for four times and filtration, the filtrate was concentrated at 90°C and dried to a constant weight by vacuum freeze dryer. The extract was 66.0413 g and the yield of the extract was 41.25% (w/w) (Hou et al., 2016). The content of CGA in EFEE was analysis by HPLC (Supplementary Material).

### Animal Experiments and Ethics Statement

All experiments were performed on 5-week-old healthy male KM mice. These mice were purchased from Chengdu DOSSY Experimental Animals Co., Ltd. The mice were housed in wire cages at 25°C under a 12 h light-dark cycle with 70% humidity.

EFEE spermatogenic activity assay. Fifty mice were divided into five groups: a negative control group, three experimental groups and a positive control group. The negative control group was intragastrically administered with water, the experimental groups were intragastrically administered with 0.4, 0.8 and 1.2 g/kg EFEE, and the positive control group was intraperitoneally injected with 5 mg/kg testosterone propionate. Each group of mice was given intragastric administration or injection once a day for 10 days.

CGA spermatogenic activity assay. The experimental method was the same as above. The experimental groups were intragastrically administered with 20, 40 and 80 mg/kg CGA.

After 10 days of administration, the mice were anaesthetized, the serum isolated from blood that was collected through heart punctures, and the testes and epididymides were collected. Testosterone and FSH were detected by ELISA kits (Shanghai Enzyme-Linked Biology Company Shanghai, China) according to the instructions. The testes were carefully removed and fixed with 10% formalin solution. After dehydration procedures, the sections were embedded in paraffin. The tissues were after that cut (with a microtome) to produce 4–5 μm sections, transferred to slides and subsequently stained using the traditional hematoxylin and eosin (H&E) stain. The testicular organ coefficient was calculated using the following formula: Testicular organ coefficient = Testicular weight/Body weight. The sperm were collected from the cauda epididymides. The cauda epididymides were minced with scissors and placed in 5 ml of 0.9% saline at 37°C for 30 min. After filtration, 10 μl aliquots of these mixtures were placed on a hemocytometer, and sperm numbers were determined by counting under an optical microscope (Park and Han, 2013; El-Khadragy et al., 2021).

### FACS Analysis of Cell Cycle

For all experiments, logarithmic growth phase C18-4 cells were plated in six-well cell culture plates and treated with 0, 10, 50 and 100 μM CGA for 36 h. Logarithmic growth phase TM3 cells were plated in six-well cell culture plates and treated with 0, 0.5, 1 and 10 μM of CGA for 36 h. Then, the cells were resuspended as single cells, washed in precooled PBS and incubated using a Cell Cycle Kit (LianKeBiology, Hangzhou, China) with 1 ml DNA staining solution and 10 μl permeabilization solution for 30 min. Cell cycle analysis was performed with a flow cytometer (Cao et al., 2012).

### Quantitative Real-Time PCR (qRT–PCR)

Total RNA was reverse-transcribed to cDNA using the HiScript III RT SuperMix reverse transcriptase reagent kit according to the reagent manual (DiNing, Beijing, China). qRT–PCR was performed on a CFX96 real-time PCR detection system (Bio–Rad, CA 94547) according to the manual for the ChamQTM Universal SYBR qPCR Master Mix kit (DiNing, Beijing, China). For more details on the qRT–PCR protocol, please refer to Wu et al. (2013). The relative expression levels of target genes and differentially expressed miRNAs were normalized to Gapdh expression for each sample respectively. The relative expression levels were calculated using 2^−ΔΔCt^ (Wu et al., 2014). The verified primers of mRNAs are listed in Supplementary Table S1.

### Cell Transfection

TM3 and C18-4 cells were transfected with shRNA (*Shp2*-mus-405: 5ʹ-GCT​GAA​CTG​GTT​CAG​TAT​TAC​TTC​A AGA​GAG​TAA​TAC​TGA​ACC​AGT​TCA​GCT​T-3ʹ) and NC (5ʹ-TTC​TCC​GAA​CGT​GTC​ACG​TTT​CAA​GAG​AAC GTG​ACA​CGT​TCG​GAG​AAT​T-3ʹ) (Genepharma Co., Shanghai, China) in a 24-well plate. After 24 h, two kinds of transfected cell were respectively treated with G418 (400 μg/ml) and hygromycin (100 μg/ml). The stably transfected cell clusters were screened by drugs and cultured. These cells were detected by qRT–PCR and Western blot.

### Immunofluorescence Staining

The protocol of immunofluorescence staining was performed according to Yu et al. (2014). Briefly, C18-4 cells and *Shp2* knockdown cells, which were cultured in a 48-well plate after treatment with CGA, were fixed with 4% formaldehyde for 15 min and washed with PBS three times for 3 min each. The cells were permeabilized with by 0.1% Triton X-100 (Solarbio, Beijing, China) for 15 min and blocked for 30 min with 1% BSA at 37°C. Then, the cells were incubated with primary antibodies specific against PLZF (1:200, Santa Cruz Biotechnology, California), SHP2 (1:100, Abways, Shanghai, China) and C-KIT (1:300, Biolegend, San Diego) for 12 h at 4°C. Alexa-488 (1:500, Beyotime Biotechnology, Shanghai, China) secondary antibodies were used to incubate cells for 1 h at 37°C. The negative control was stained with conjugated secondary antibodies alone: goat anti-rabbit IgG and goat anti-mouse IgG. The nuclei of cells were stained with DAPI (Beyotime Biotechnology, Shanghai, China) (Niu et al., 2016).

### Western Blot Analysis

The cells were treated with different doses of CGA for 36 h. The proteins were extracted using a protein extraction reagent kit (Solarbio, Beijing, China). Protein quantification was performed using a BCA quantification kit (Solarbio Beijing, China). 10% separation gel was prepared. Each well was loaded with 30 μg of the protein. The denatured proteins were separated by SDS–PAGE (80 V, 30 min; 120 V, 60 min) and transferred onto PVDF membranes (80 V, 135 min). The membranes were blocked for 2 h at 37°C in TBST containing 5% skim milk and then incubated for 12 h at 4°C in TBST containing specific primary antibodies (SHP2, ERK1/2, pERK1/2, StAR, or β-actin, 1:1000) (Abways, Shanghai, China). After four washes with TBST, the membranes were incubated with HRP-conjugated secondary antibody (Solarbio Beijing, China) for 1.5 h at 37°C. After washing the membranes with TBST three times, the signals were visualized using ECL (DiNing, Beijing, China), and the membranes were exposed on X-ray films. The quantification of protein bands were analyzed by ImageJ version 1.51.

### Network Pharmacology Analysis

The main components and their related genes in EF were collected by summarizing the research work on chemical components of EF (He et al., 2014; Wang C.Y. et al., 2019) and searching TCMSP (Ru et al., 2014) (http://tcmspnw.com), Pharm Mapper (Wang et al., 2017) (http://www.lilab-ecust.cn/pharmmapper/) and other traditional Chinese medicine components databases. The selected genes were analysed by literature research (Paz et al., 2016; Puri and Walker, 2016; Zhou et al., 2016), Kegg (Kanehisa et al., 2017) (https://www.kegg.jp/kegg/) and KOBAS (http://kobas.cbi.pku.edu.cn/) enrichment to investigate the possible biological functions of the potential targets and the biological pathways involved in spermatogenesis. Cytoscape version 3.5.1 was used to draw the network diagram of “component-target-pathway-function” (Shannon et al., 2003; Killcoyne et al., 2009) (Supplementary Material).

### Statistical Analysis

Data analysis was performed using IBM SPSS statistical software (version 23.0). One-way analysis of variance with a *post hoc* test was used for multiple comparisons. The histograms were drawn using GraphPad Prism 7. The results were considered significant or extremely significant at a level of *p* < 0.05 or *p* < 0.01.

## Results

### The Effects of EF Extracts on Spermatogenesis in Mice

**FIGURE 1:**
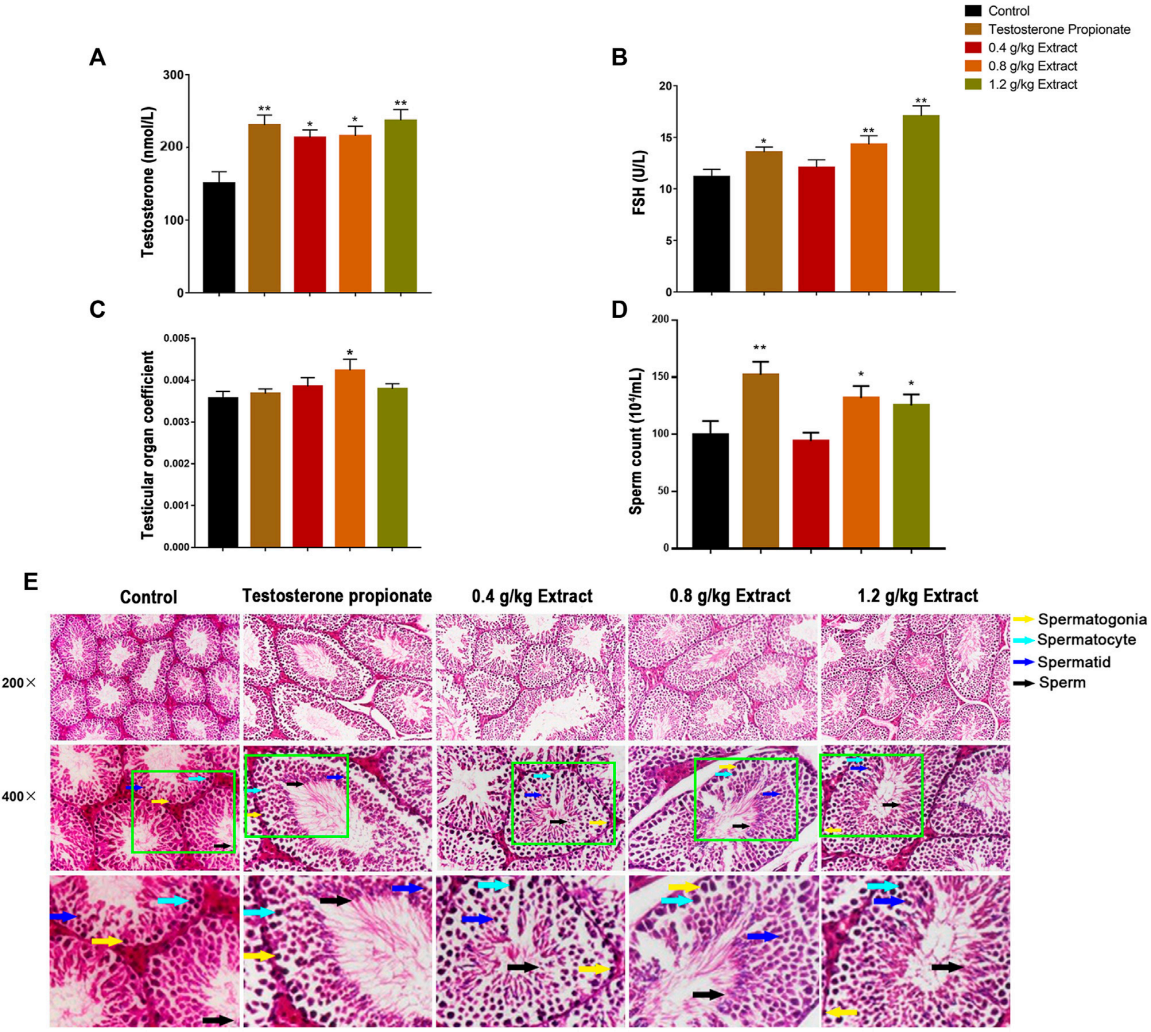
The effects of EF extracts on spermatogenesis in mice (Control: water; Testosterone Proplonate: 5 mg/kg; Extracts: 0.4, 0.8, 1.2 g/kg). **(A)** The concentration of serum testosterone was analysed by ELISA. **(B)** The concentration of serum FSH was analysed by ELISA. **(C)** Testicular organ coefficient analysis of testes. **(D)** The sperm count analysis of epididymis. **(E)** Haematoxylin and eosin (HE) staining analysis of testes treated with different doses of EFEE. Values are expressed as the mean ± SD (*n* = 10).*p < 0.05, **p < 0.01 vs control.

As shown in Figures 1A,B, compared with the control group, the serum testosterone and FSH levels of 0.8 and 1.2 g/kg EFEE groups were significantly increased (*p* < 0.01, *p* < 0.05). Similarly, the testicular tissue coefficient of mice in the medium-dose groups was significantly higher (*p* < 0.05) than that in the control group (Figure 1C). The sperm count of the 0.8 and 1.2 g/kg EFEE groups were higher (*p* < 0.05) than that in the control group (Figures 1D,E). Therefore, EFEE could promote the secretion of spermatogenic hormones and sperm production in mice.

### Network Pharmacological Analysis of EF Active Components and Their Mechanism

**FIGURE 2:**
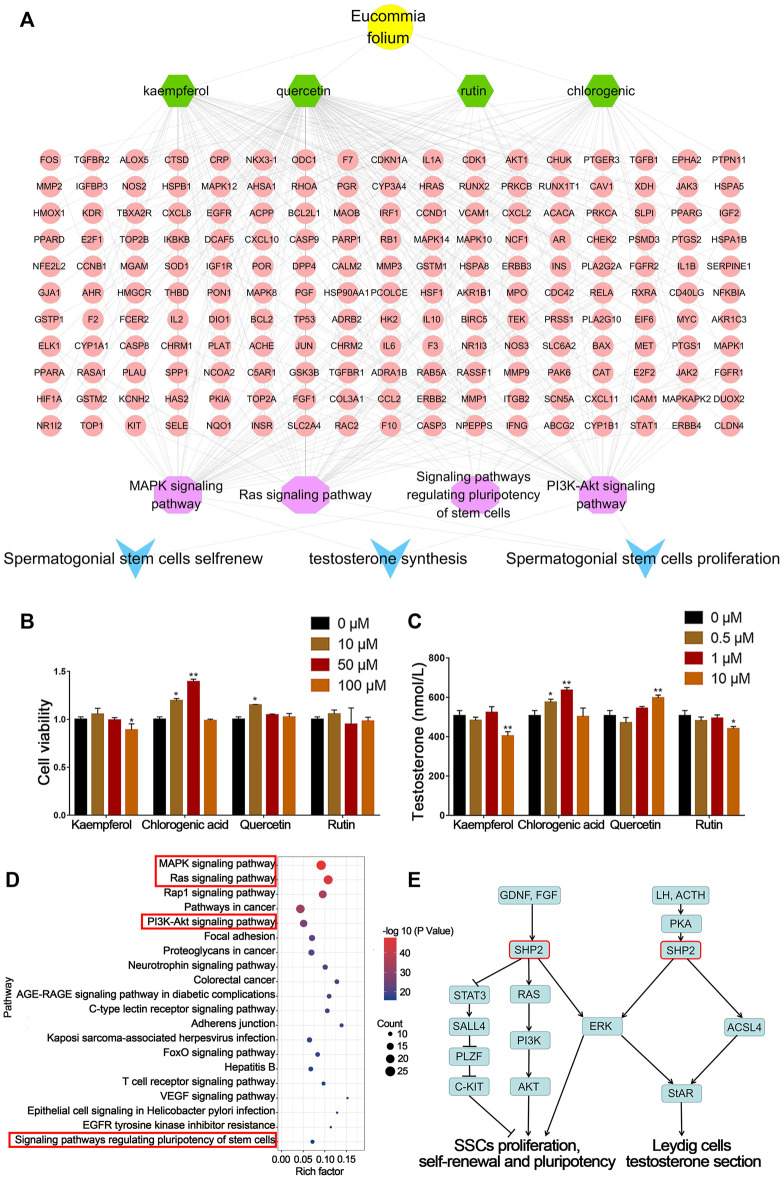
Network pharmacology prediction and validation of spermatogenesis related compounds in EF. **(A)** Network pharmacology analysis of EF active compounds and their mechanism. **(B)** C18-4 cell viability. **(C)** Testosterone secretion in TM3 cells. **(D**,**E)** KEGG pathway analysis of putative target genes of CGA. Values are expressed as the mean ± SD (*n* = 3). *p < 0.05, **p < 0.01 vs 0 μM.

Through network pharmacological analysis, chemical compounds in EF related to spermatogenesis mainly involved MAPK, RAS, PI3K-Akt and regulating the pluripotency of the stem cell signaling pathway. These signaling pathways were involved in the biological processes of SSC proliferation, self-renewal and Leydig cell testosterone secretion. The corresponding compounds were screened to obtain quercetin, kaempferol, CGA and rutin. The targets of these compounds were mainly concentrated in four signaling pathways (Figure 2A). These results suggested that quercetin, kaempferol, CGA and rutin were the main active components in EF that may be involved in spermatogenesis.

### Effects of Screened Compounds on Spermatogenesis

As shown in Figure 2B, the four compounds screened above were tested for their biological activities in promoting SSC proliferation and testosterone secretion. Compared with the control group (0 μM), C18-4 cell proliferation in the CGA groups (10 and 50 μM) below their safe concentrations was significantly increased (*p* < 0.05, *p* < 0.01). In addition, quercetin treatment (10 μM) also significantly promoted cell proliferation (*p* < 0.05).

As shown in Figure 2C, the CGA (0.5 and 1 μM) and quercetin (10 μM) treatment groups also significantly enhanced (*p* < 0.01, *p* < 0.05) testosterone secretion in TM3 cells, and the CGA groups exhibited stronger activity at low concentrations. The content of CGA in EFEE was 2.58% (w/w) by HPLC (Supplementary Figure S1). These results indicated that CGA was the main compound of EFEE and involved in spermatogenesis. Therefore, CGA was selected for further experiments.

### The Effects of CGA on Spermatogenesis in Mice

**FIGURE 3:**
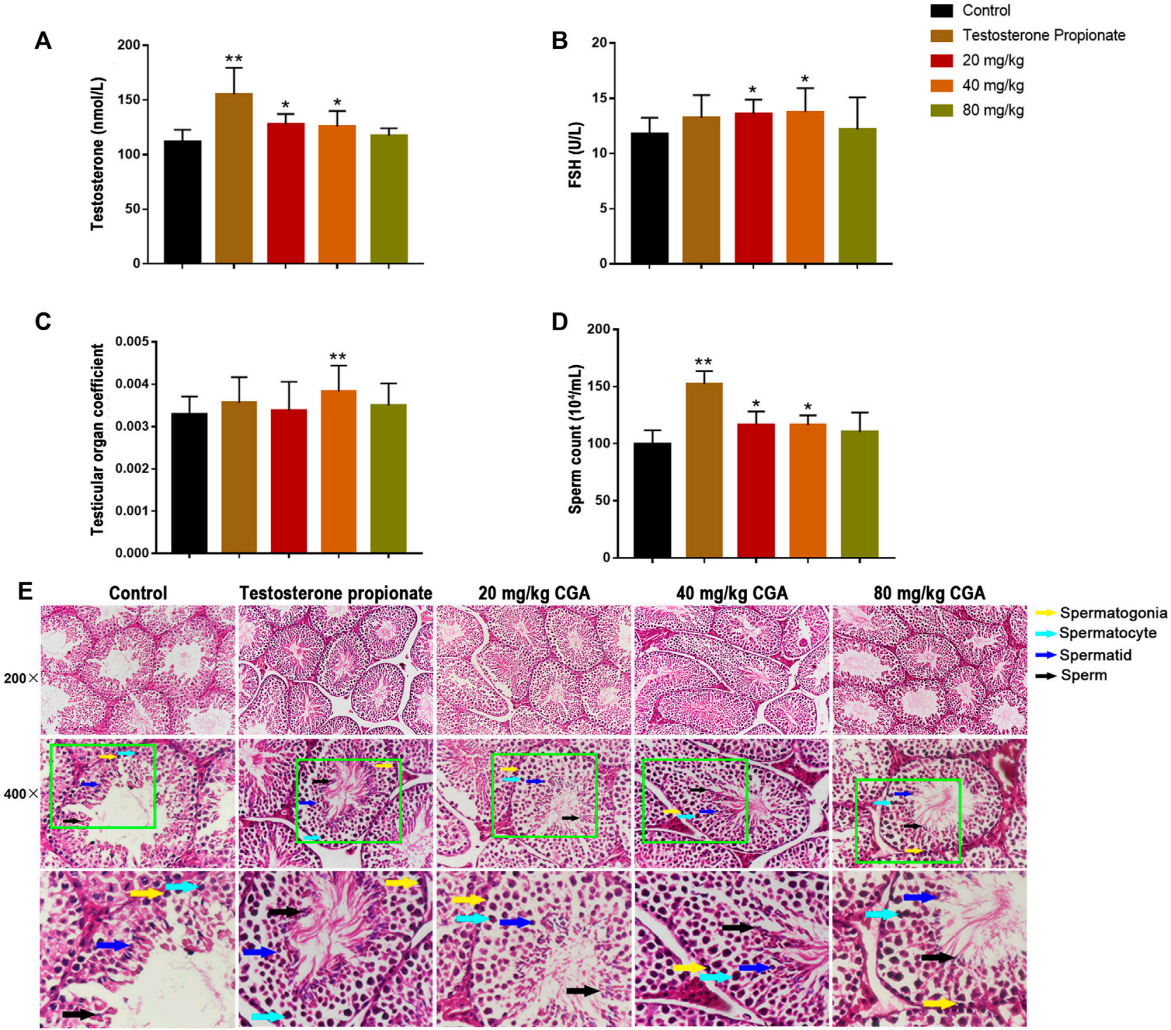
The effects of CGA on spermatogenesis in mice (Control: water; Testosterone Proplonate: 5 mg/kg; CGA: 20, 40, 80 mg/kg). **(A)** The concentration of serum testosterone was analysed by ELISA. **(B)** The concentration of serum FSH was analysed by ELISA. **(C)** Testicular organ coefficient analysis of testes. **(D)** The sperm count analysis of epididymis. **(E)** Haematoxylin and eosin (HE) staining analysis of testes treated with different doses of EFEE. Values are expressed as the mean ± SD (*n* = 10).*p < 0.05, **p < 0.01 vs control.

In order to determine the effect of CGA on spermatogenesis *in vivo*, the mice were intragastrically administered with 20, 40 and 80 mg/kg CGA. As shown in Figures 3A,B, compared with the control group, the serum testosterone and FSH levels in groups treated with 20 and 40 mg/kg of CGA were significantly increased (*p* < 0.05). Similarly, the testicular tissue coefficient of mice in the medium-dose groups was significantly higher (*p* < 0.05) than that in the control group (Figure 3C). The sperm count of 20 and 40 mg/kg CGA groups were higher (*p* < 0.01, *p* < 0.05) than that in the control group (Figures 3D,E). The results suggested that CGA could promote the secretion of spermatogenic hormones and sperm production in mice.

### The Signaling Pathways of CGA Regulate Spermatogenesis by Network Pharmacology Analysis

KEGG enrichment analysis showed that CGA might affected the SSC proliferation, self-renewal, and leydig cell testosterone secretion by regulating MAPK, RAS, PI3K-Akt and Pluripotency of stem cells signaling pathways (Figure 2D). As shown in Figure 2E, SHP2 was involved in these signaling pathways above. These results indicated that *Shp2* is a key gene in CGA regulation of spermatogenesis. Therefore, the subsequent studies mainly focused on the regulation of CGA on the expression of SHP2 and its downstream genes.

### The Effect of CGA on SSC Proliferation

**FIGURE 4:**
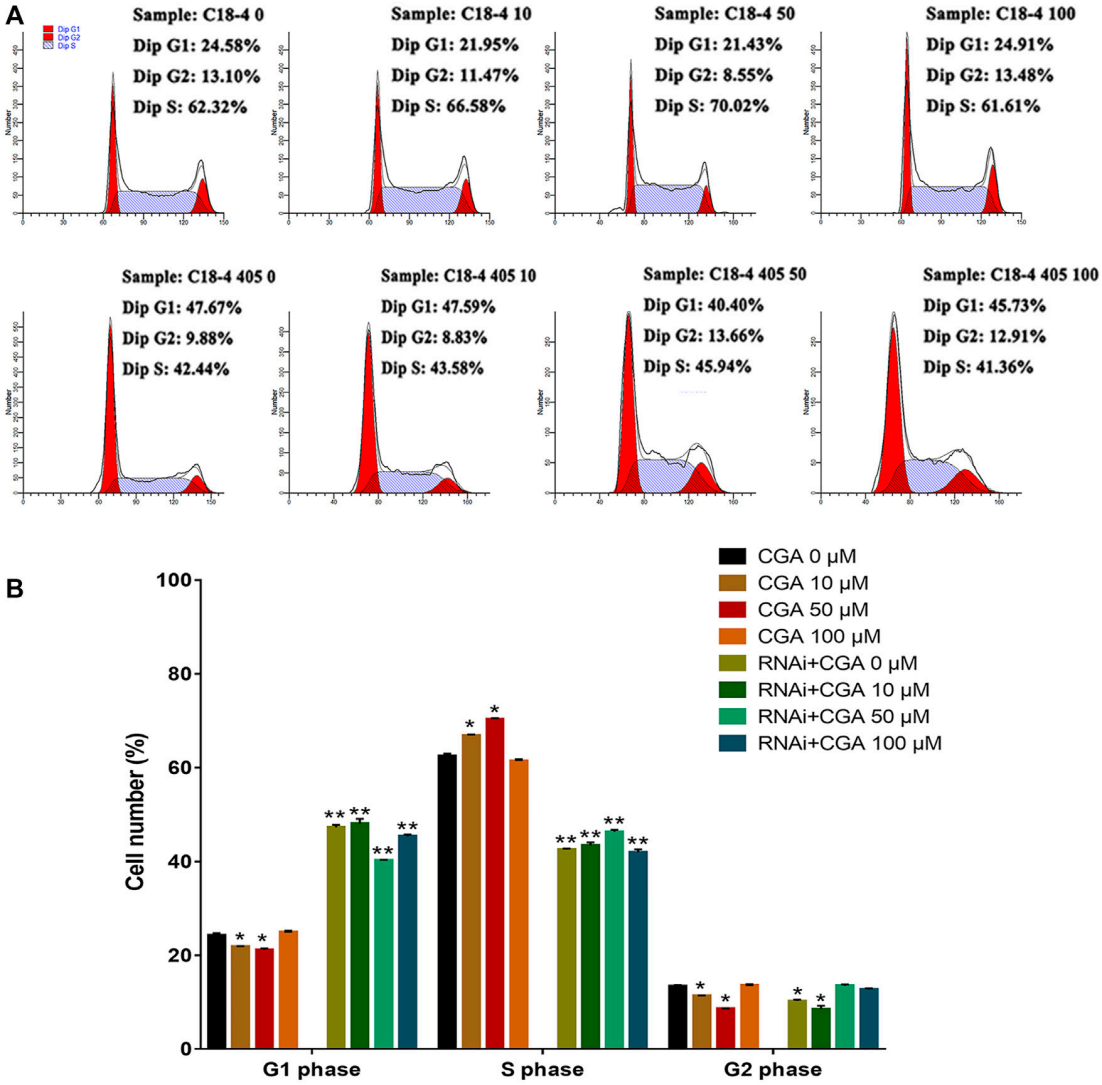
The effect of CGA on SSCs proliferation. C18-4 cells and *Shp2* knockdown C18-4 cells (RNAi) were cultured with CGA (10, 50, and 100 μM) for 36 h, and the cell cycle was determined using FACS. **(A)** FACS analysis of the cell cycle. **(B)** The results of statistical analyses. Values are expressed as the mean ± SD (*n* = 3)*p < 0.05, **p < 0.01 vs CGA 0 μM.

SHP2 is a widely expressed protein tyrosine phosphatase that is necessary for signal transduction from multiple cell surface receptors (Puri and Walker, 2016). Compared with the control group (0 μM), CGA (10 and 50 μM) significantly increased (*p* < 0.05) the S phase and reduced the G1 phase of the C18-4 cell cycle (Figures 4A,B). This indicated that CGA could enhance the C18-4 cells proliferation. The inhibition of SHP2 expression significantly decreased the proportion of cells in S phase and increased the proportion of cells in G1 phase (*p* < 0.01). Moreover, CGA had no significant effect on the cell cycle of *Shp2* knockdown C18-4 cells. These results suggested that the *Shp2* gene plays a key role in the promotion of C18-4 cells proliferation by CGA.

### CGA Activated the SHP2-MAPK Signaling Pathway

**FIGURE 5:**
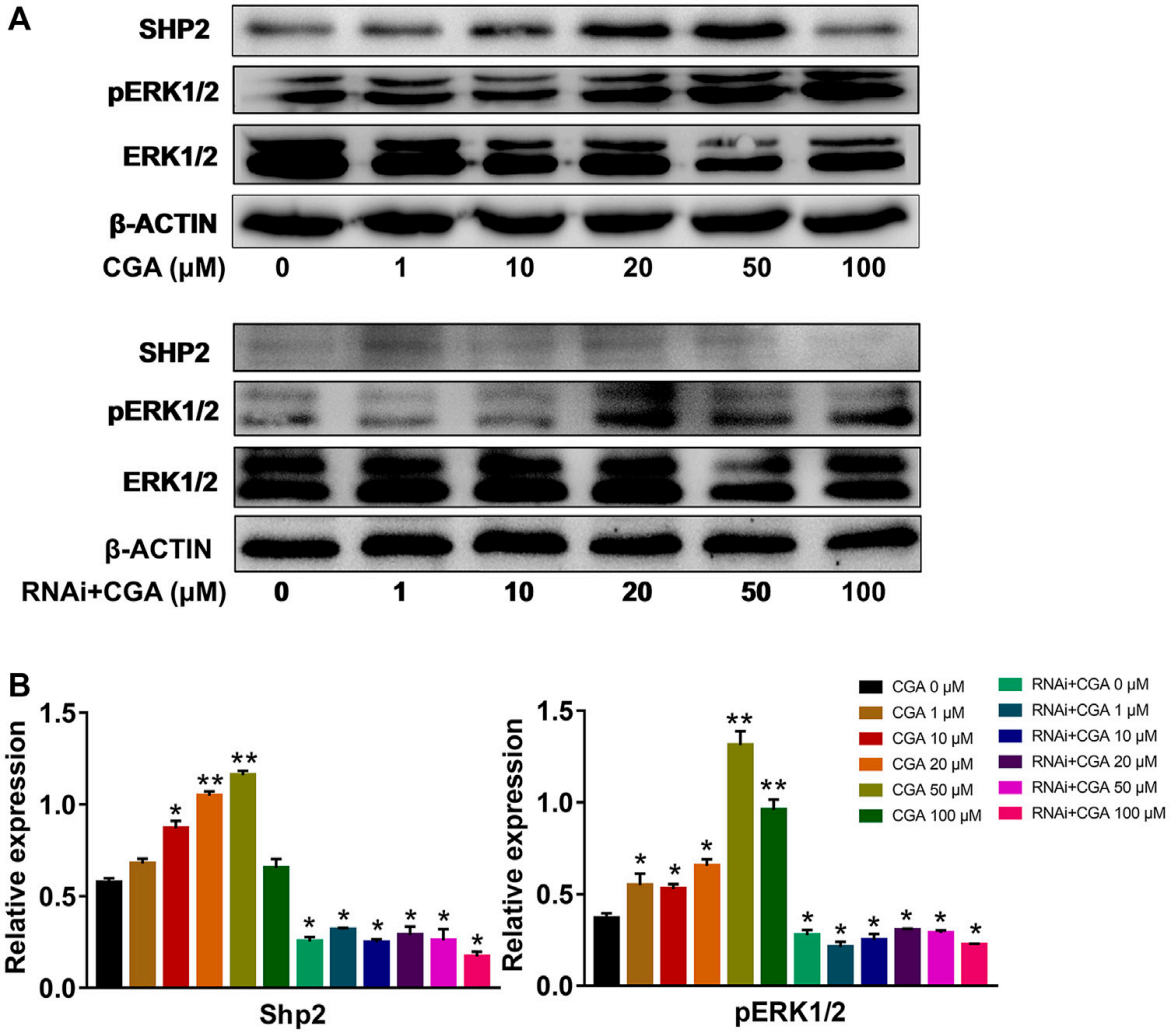
CGA activated SHP2-MAPK signaling pathway. C18-4 cells and *Shp2* knockdown C18-4 cell (RNAi) were cultured with CGA (1, 10, 20, 50 and 100 μM) for 36 h, and gene expression was determined using Western blotting. **(A)** Western blot analysis of the effect of CGA on the SHP2-MAPK signaling pathway. **(B)** The results of statistical analyses. Values are expressed as the mean ± SD (*n* = 3) *p < 0.05, **p < 0.01 vs CGA 0 μM.

The SHP2-MAPK signaling pathway is required for maintaining SSC proliferation and self renewal, and spermatogenesis (Puri et al., 2014). As shown in Figures 5A,B, CGA (1, 10, 20 and 50 μM) treatment induced the SHP2 expression (*p* < 0.05, *p* < 0.01) and ERK1/2 phosphorylation (*p* < 0.05, *p* < 0.01) in C18-4 cells. After inhibiting *Shp2* gene expression, ERK1/2 phosphorylation was significantly reduced compared with that in the control group (0 μM). Meanwhile, CGA could not significantly promote ERK1/2 phosphorylation in *Shp2* knockdown cells. These results indicated that CGA could induce SHP2-MAPK signaling pathway activation in SSCs.

### The Effect of CGA on SSC Self-Renewal

**FIGURE 6:**
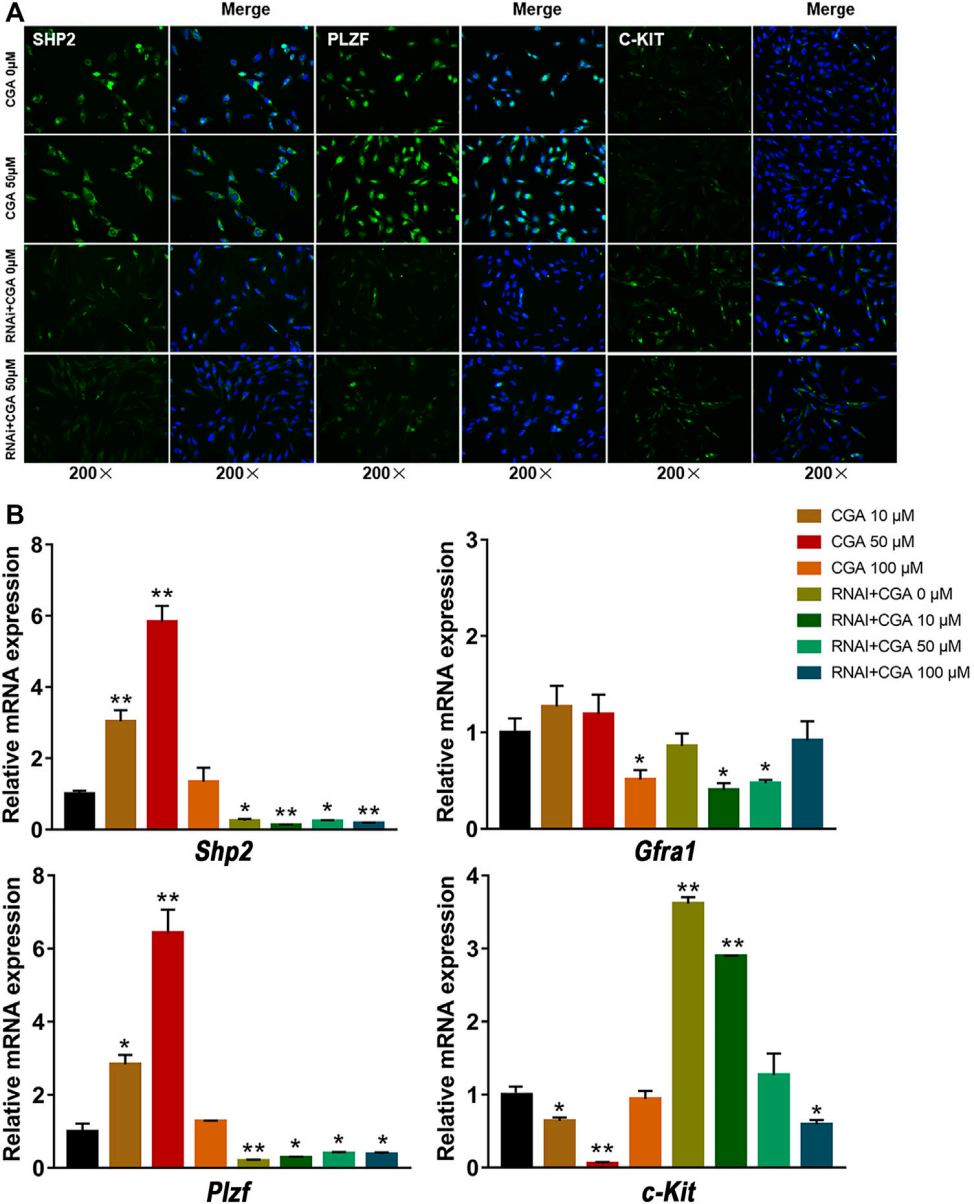
The effect of CGA on SSC self-renewal. C18-4 cells and *Shp2* knockdown C18-4 cells (RNAi) were cultured with CGA (10, 50, and 100 μM) for 36 h, and gene expression was measured by immunofluorescence immunofluorescence staining and qRT–PCR. **(A)** Immunofluorescence staining analysis of the effect CGA on SHP2, PLZF and C-KIT expression. **(B)** qRT–PCR analysis of the effect of CGA on *Shp2*, *Plzf*, *c-Kit* and *Gfra1* expression. Values are expressed as the mean ± SD (*n* = 3) *p < 0.05, **p < 0.01 vs CGA 0 μM.

SSCs play an essential role in maintaining highly productive spermatogenesis by self-renewal and the continuous generation of daughter spermatogonia that differentiate into spermatozoa, transmitting genetic information to the next generation (Subash and Kumar, 2021). GFRA1 and PLZF are required for the regulation of SSC self-renewal (Buaas et al., 2004; Costoya et al., 2004), and C-KIT is a critical factor in SSC differentiation (Zhang et al., 2014). As shown in Figures 6A,B, the C18-4 cells treated with CGA (10 and 50 μM) induced the expression of SHP2 and PLZF, but decreased C-KIT expression (*p* < 0.05, *p* < 0.01). The inhibition of SHP2 expression reduced PLZF expression and promoted C-KIT expression (*p* < 0.05, *p* < 0.01). These results suggested that CGA could promote the self-renewal of C18-4 cells and maintain their homeostasis.

### Effect of CGA on Testosterone Secretion in Leydig Cells

**FIGURE 7:**
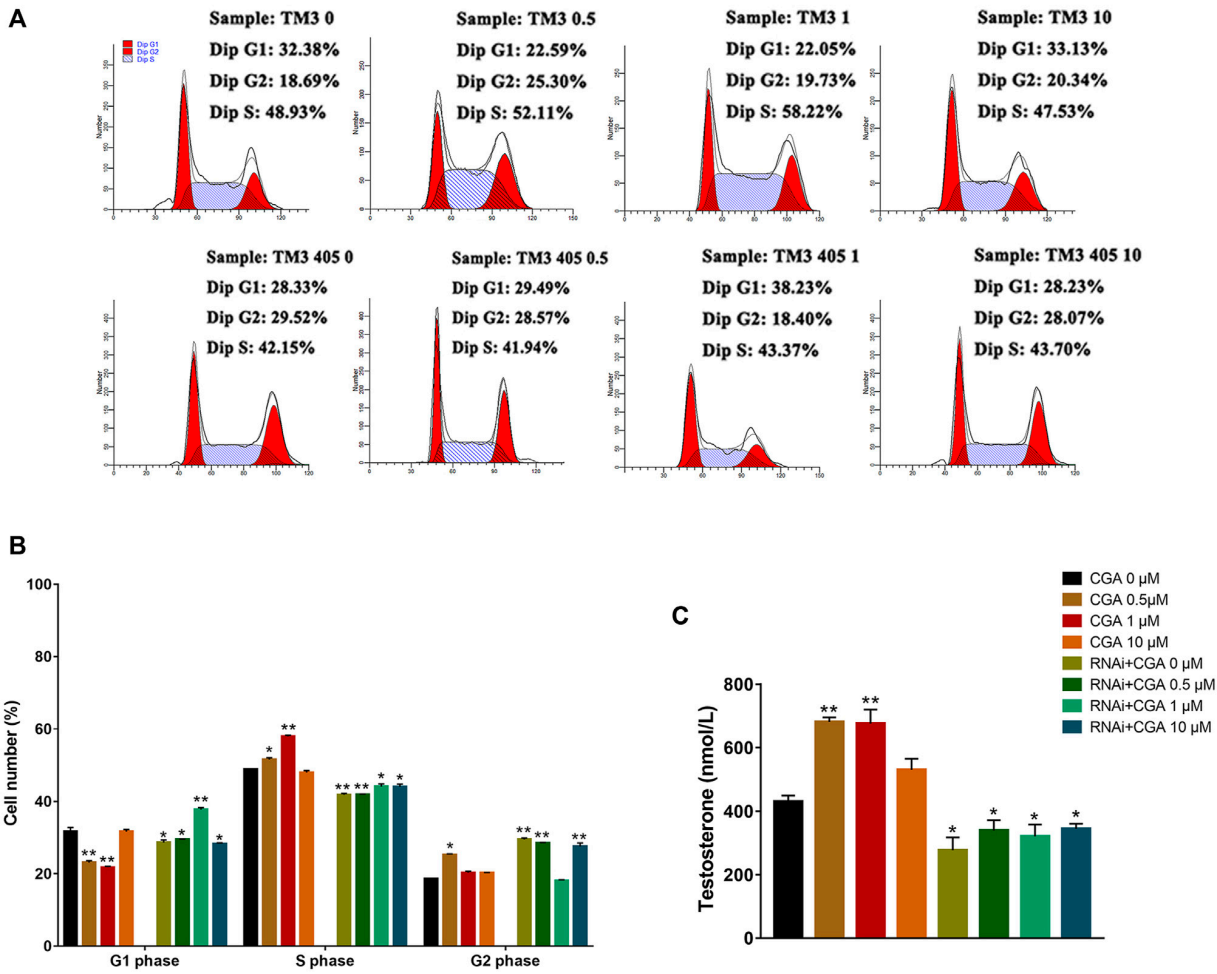
Effect of CGA on testosterone secretion in leydig cells. TM3 cells and *Shp2* knockdown TM3 cells (RNAi) were cultured with CGA (0.5, 1, and 10 μM) for 36 h; the cell cycle was measured by FACS, and testosterone secretion was measured by ELISA. **(A)** FACS analysis of the cell cycle. **(B)** The results of statistical analyses. **(C)** ELISA analysis of the effect of CGA on testosterone secretion. Values are expressed as the mean ± SD (*n* = 3). *p < 0.05, **p < 0.01 vs CGA 0 μM.

Compared with the control group, CGA (0.5 and 1 μM) effectively promoted testosterone secretion in TM3 cells (*p* < 0.01). Testosterone secretion in TM3 cells was significantly reduced after the *Shp2* gene was inhibited (*p* < 0.05) (Figure 7C). Furthermore, CGA (0.5 and 1 μM) also significantly increased the proportion of TM3 cells in the S phase of the cell cycle (*p* < 0.01, *p* < 0.05). The knockdown of *Shp2* gene expression significantly reduced the proportion of cells in S phase compared with that in the control group (*p* < 0.01, *p* < 0.05) (Figures 7A,B). These results suggested that CGA could promote testosterone secretion and proliferation in TM3 cells.

### CGA Increased the Expression of StAR

**FIGURE 8:**
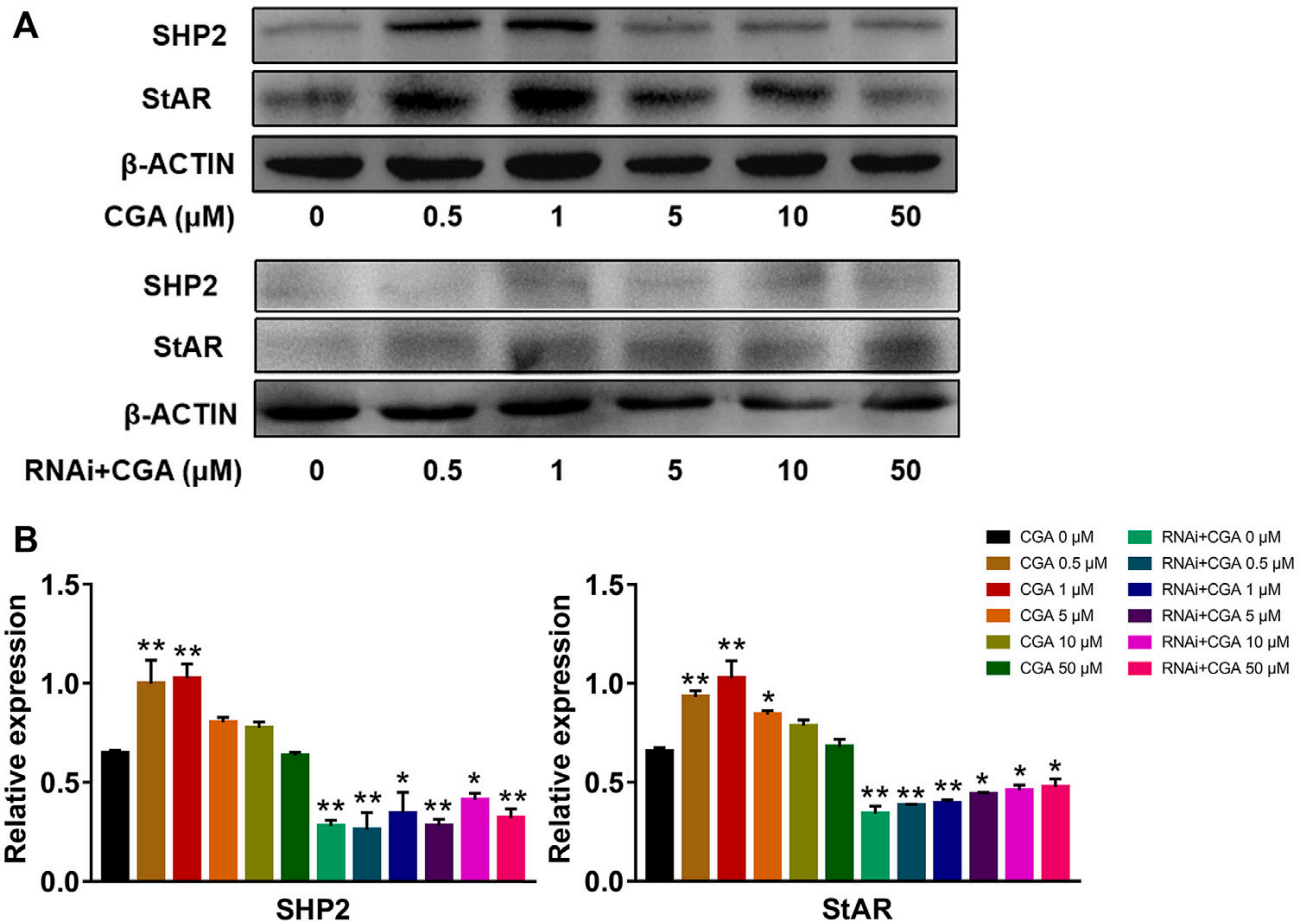
CGA activated the SHP2-StAR signaling pathway. TM3 cells and *Shp2* knockdown TM3 cells (RNAi) were cultured with CGA (0.5, 1, 5, 10 and 50 μM) for 36 h, and gene expression was measured by Western blot. **(A)** Western blot analysis of the effect of CGA on the SHP2-StAR signaling pathway. **(B)** The results of statistical analyses. Values are expressed as the mean ± SD (*n* = 3). *p < 0.05, **p < 0.01 vs CGA 0 μM.

SHP2 regulates the expression of the downstream *Star* gene, which is a rate-limiting enzyme involved in testosterone secretion in leydig cells (Cooke et al., 2011). As shown in Figures 8A,B, CGA (0.5 and 1 μM) induced SHP2 expression (*p* < 0.01) and promoted StAR expression in TM3 cells (*p* < 0.01). The inhibition of *Shp2* gene expression significantly reduced the expression of StAR compared with that in the control group (*p* < 0.01, *p* < 0.05), and CGA did not promote *Star* gene expression in *Shp2* knockdown TM3 cells. These results suggested that CGA could promote the testosterone secretion by improving the expression of SHP2 and StAR.

## Discussion

The current study demonstrated that EFEE could promote the spermatogenesis and serum testosterone levels in mice. These changes induced by EFEE may be explained by CGA promoting the proliferation of SSCs and testosterone secretion in leydig cells. Moreover, CGA could promote the spermatogenesis and serum testosterone levels *in vivo.* The promotion of SSC proliferation was induced by the activation of the SHP2-MAPK signaling pathway after treatment with CGA. Meanwhile, CGA significantly enhanced the expression of SHP2 and StAR, and induced testosterone secretion in leydig cells.

Spermatogenesis is a very complicated process that requires the involvement of a variety of cells, hormones, paracrine factors, genes and epigenetic regulators (Neto et al., 2016). As the origin of spermatogenesis, SSCs were located at the base of seminiferous tubules in the testes of male animals. Most SSCs die during the process of spermatogenesis, but one SSC can still produce hundreds of sperm (Kanatsu-Shinohara et al., 2019), so hundreds of millions of sperm can be found in the testis (Law et al., 2019). The development of SSCs into mature sperm is regulated by a variety of proteins and hormones. Testosterone, which is secreted by leydig cells in the testis, is required for at least four critical processes during spermatogenesis: meiosis, sertoli-spermatid adhesion, maintenance of the BTB and sperm release (Smith and Walker, 2014). These studies indicate that the proliferation of SSCs and testosterone secretion in leydig cells are critical processes in spermatogenesis. Therefore, the SSC proliferation and testosterone secretion were selected as the conditions for studying the active spermatogenic compounds of EF.

In recent years, network pharmacology has been widely used in the mechanism of TCM research as a method to predict compounds of TCM and their targets (Luo et al., 2020). In order to investigate the mechanism of which EF promotes spermatogenesis, network pharmacology was used to predict the active compounds in EF. CGA, quercetin, rutin, and kaempferol in EF were predicted to be related to SSC proliferation and testosterone secretion, and the main component CGA was screened to have strong activity. Meanwhile, combined with previous literature reports (Paz et al., 2016; Puri and Walker, 2016; Zhou et al., 2016) and the network pharmacology analysis showed that *Shp2* might be a key gene related in the CGA regulation of spermatogenesis. Furthermore, HPLC result showed that the content of CGA in EFEE was more than 2.58% (w/w) (Supplementary Figure S1). These results indicate that CGA was the main active component of EFEE in promoting spermatogenesis. Previous studies showed that CGA could increase the number of sperm in rat testis, moreover, CGA abated tamoxifen-mediated reproductive toxicities and improved the testosterone secretion and sperm motility in male rats (Park and Han, 2013; Owumi et al., 2021). These studies indicate that CGA could promote the spermatogenesis, and our study was consistent with results above. CGA can not only promote the proliferation of various cells but also inhibit the apoptosis of cells (Zhang and Hu, 2016; Liu and Li, 2021). These biological functions of CGA play an important role in spermatogenesis.

In germ cells of the testis, Src homology phosphotyrosyl phosphatase 2 (SHP2) is expressed in most immature A-single spermatogonia (As, SSC) and A-aligned (A_al_) spermatogonia cells (Puri and Walker, 2016). Studies have shown that spermatogenic cells at all levels of the tubules were absent after *Shp2* knockout. This result suggested that SHP2 is essential for SSCs to maintain fertility (Hu et al., 2015; Puri et al., 2014). Moreover, SHP2 could also promote the activation of the MAPK signaling pathway during SSC proliferation (Li et al., 2020). CGA, the main active compound in EF, could promote the expression of the *Shp2* gene (Zhou et al., 2016). The results of the present study were consistent with these studies. In addition, the phosphorylation level of ERK1/2 in SSCs treated with CGA was significantly increased and further promoted their proliferation. After *Shp2* knockdown, ERK1/2 phosphorylation was significantly reduced and cell proliferation was inhibited. Moreover, CGA had no significant effect on ERK1/2 phosphorylation or the cell cycle in the *Shp2* knockdown SSCs. These results suggested that CGA could enhance the phosphorylation of ERK1/2 by promoting *Shp2* expression and further accelerate SSC proliferation.

Approximately 35,000 SSCs are present in a mouse testis, comprising approximately about 0.035% of all germ cells (Tegelenbosch and de Rooij, 1993). In rodents, stem cell activity is exhibited in a subpopulation of undifferentiated spermatogonia that are present as A_s_. A_s_ cells can divide to produce separated A_s_ cells that retain stem cell activity, or they can divide to produce pairs (A_pr_) and undifferentiated A_al_ spermatogonia that increasingly lose stem cell activity (Nakagawa et al., 2010; Hara et al., 2014). A_al_ cells proliferate further by mitotic divisions and enter a differentiation program that results in the formation of preleptotene spermatocytes that undergo meiosis to produce haploid round spermatids that mature and elongate to form spermatozoa. Markers of undifferentiated spermatogonia that are conserved from rodents to nonhuman primates to humans include GFRa1, UTF1, PLZF, SALL4 and LIN28. C-KIT is a conserved marker of differentiated spermatogonia (Fayomi and Orwig, 2018). In the present study, we detected the differentiation of SSCs by analysing mRNAs and proteins of the marker genes *Gfra1*, *Plzf* and *c-Kit*. The result indicated that, in both of mRNA and protein levels, CGA could promote the expression of PLZF and that C-KIT was inhibited. After *Shp2* knockdown, the PLZF expression was significantly decreased and C-KIT was promoted. These results suggested that CGA inhibits SSC differentiation and maintains self-renewal by regulating SHP2.

As one of the important sex hormones in mammals, testosterone plays an important role in maintaining the normal reproductive function of males. Testosterone affects sex differentiation in the embryonic stage after initial oestrus, regulates spermatogenesis and maintains male secondary sexual characteristics (Smith and Walker, 2014; Skurikhin et al., 2017; Rey, 2021). StAR assists cholesterol in entering the mitochondria and this process is a rate-limiting step in testosterone secretion (Hasegawa et al., 2000). In this study, CGA induced the expression of SHP2 and StAR in leydig cells and further enhanced testosterone secretion. After *Shp2* gene knockdown, CGA did not promote StAR expression or testosterone secretion. In conclusion, CGA could promote the expression of StAR through SHP2 and further enhance the transport of cholesterol to improve the secretion of testosterone.

By flow cytometry, we found that CGA could promote the proliferation of leydig cells, while cell proliferation was inhibited with the knockdown of *Shp2*. These results indicated that the effect of CGA on promoting the proliferation of leydig cells were related to the *Shp2* gene, but the specific mechanism is still unclear, and is worthy of further study.

## Conclusion

In this study, we found that EF could promote the spermatogenesis of mice *in vivo*. Furthermore, network pharmacological prediction and bioactivity screening were applied to obtain the main compounds that could promote spermatogenesis. In subsequent studies, CGA, one of the main spermatogenic active compounds of EF, could promoted spermatogenesis *in vivo*, and promote SSC proliferation and Leydig cell testosterone secretion through *Shp2* gene-mediated corresponding signaling pathways. Our study provided strong evidence for elucidating the mechanism by which EF promotes spermatogenesis in mice and a new theoretical basis for dealing with the decrease in male reproductive capacity.

## Data Availability

The original contributions presented in the study are included in the article/Supplementary Material, further inquiries can be directed to the corresponding author.
