# Supplementary material for: Study on the SHP2-Mediated Mechanism of Promoting Spermatogenesis Induced by Active Compounds of Eucommiae Folium in Mice
Source: Front Pharmacol. 2022 Mar 22;13:851930. doi: 10.3389/fphar.2022.851930 (PMC8981153; doi:10.3389/fphar.2022.851930)
Supplement: Supplementary file 1 [file Table1.docx]

| Primers | Forward (5’–3’) | Reverse (5’–3’) |
| --- | --- | --- |
| Shp2 | TTTGATCATACCAGGGTCGTT | ATCCGCCAGAAGTCATTCACC |
| Plzf | CGCCACCTTCGCTCACATACAG | GCCACAGCCATTACACTCATAGGG |
| c-Kit | GATCTGCTCTGCGTCCTGTTGG | TGGATGGATGGATGGCGGAGAC |
| Gfra1 | GACCGCCTGGACTGTGTGAAAG | TCAGTGTGCGGTACTTGGTGC |
| Gapdh | AGGTCGGTGTGAACGGATTTG | TGTAGACCATGTAGTTGAGGTCA |
