# Supplementary figures and images for: Study on the SHP2-Mediated Mechanism of Promoting Spermatogenesis Induced by Active Compounds of Eucommiae Folium in Mice
Source: Front Pharmacol. 2022 Mar 22;13:851930. doi: 10.3389/fphar.2022.851930 (PMC8981153; doi:10.3389/fphar.2022.851930)

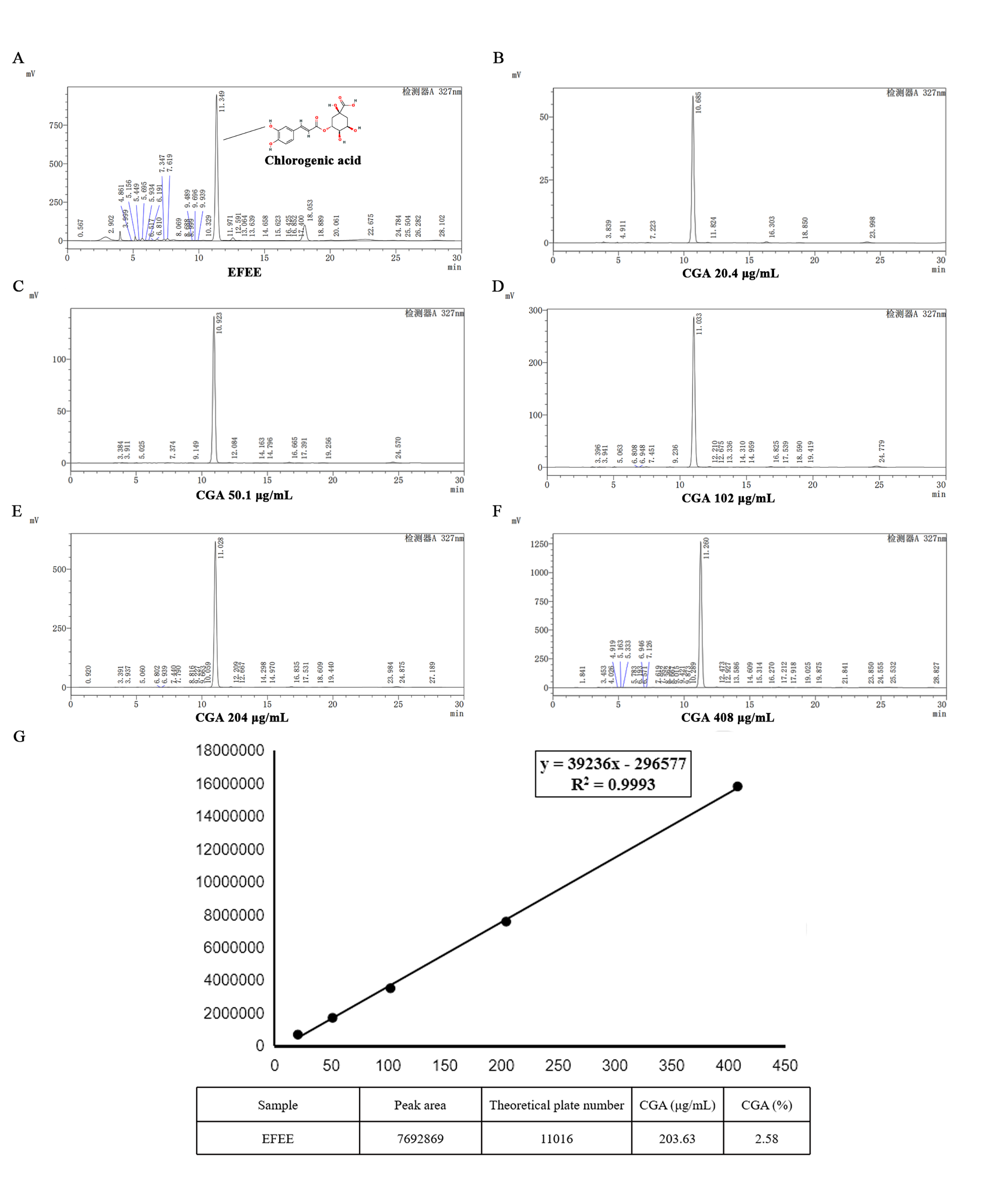

Supplement: Supplementary file 2 [file Image1.TIF]
