# Supplementary material for: Study on the SHP2-Mediated Mechanism of Promoting Spermatogenesis Induced by Active Compounds of Eucommiae Folium in Mice
Source: Front Pharmacol. 2022 Mar 22;13:851930. doi: 10.3389/fphar.2022.851930 (PMC8981153; doi:10.3389/fphar.2022.851930)
Supplement: Supplementary file 3 [file Table2.docx]

| Wavelength | Flow rate | Column temperature | Mobile phase (v/v) |
| --- | --- | --- | --- |
| 327 nm | 1 mL/min | 25 ℃ | Acetonitrile : 4% Phosphoric acid solution (13:87) |
