# Supplementary material for: Study on the SHP2-Mediated Mechanism of Promoting Spermatogenesis Induced by Active Compounds of Eucommiae Folium in Mice
Source: Front Pharmacol. 2022 Mar 22;13:851930. doi: 10.3389/fphar.2022.851930 (PMC8981153; doi:10.3389/fphar.2022.851930)
Supplement: Supplementary file 4 [file DataSheet1.docx]

Supplementary materials

**Material and methods**

2.3 HPLC assay of CGA in EFEE

EFEE (0.1975 g) was dissolved in 25 mL of 50 % (v/v) methanol solution and mixed by ultrasonic. The sample was refluxed for 30 min, at 60 ℃. After the sample was cooling to room temperature, the loss of sample weight was supplemented with 50% (v/v) methanol solution. The sample filtered through 0.45 µm syringe filters for HPLC assay. The standard curve exhibited a good linear relationship between absorbance and CGA concentration from 20.4 to 408 μg/L (R2 = 0.9993). The chromatographic conditions of HPLC are listed in Table S1 (Hou et al.,2016).

2.10 Network pharmacology

The main components and their related genes in EF were collected by summarizing the research work on chemical components of EF (He et al., 2014; Wang et al., 2019) and searching TCMSP (Ru et al., 2014) (http://tcmspnw.com), Pharm Mapper (Wang et al., 2017) (http://www.lilab-ecust.cn/pharmmapper/) and other traditional Chinese medicine components databases.

In this study, the compounds are selected out with favorable pharmacokinetics properties according to the ADME system (absorption, distribution, metabolism and excretion), whose parameters includes oral bioavailability (OB), and drug-likeness (DL). The threshold values for ADME evaluation system are OB ≥ 20%; DL ≥ 0.2 (Xu et al., 2012;Tao et al*.*, 2013). In addition, some compounds with high content and high bioavailability were supplemented by literature search.

In order to identify the targets of chemical compounds of EF, the reverse pharmacophore matching database: PharmMapper was used to hunt for targets. First, all the “.Sdf” format of the chemical structure of EF are downloaded from the PubChem database (Kim et al., 2016) (https://pubchem.ncbi.nlm.nih.gov/), and upload. Then, users select “Druggable Pharmacophore Models”, set the number of matching targets to 300, click submit to get the targets of each chemical compounds. The UniProt database (Bairoch et al., 2005) (http://www.uniprot.org/uniprot/) was used to correct official symbols of all the targets by inputting the target names and limiting the species to “Mus musculus”. Finally, the targets that do not meet the setting parameters and duplicated will be eliminated.

The selected genes were analysed by literature search, Kegg (Kanehisa et al., 2017) (https://www.kegg.jp/kegg/) and KOBAS (http://kobas.cbi.pku.edu.cn/) enrichment to investigate the possible biological functions of the potential targets and the biological pathways involved. Finally Cytoscape version 3.5.1 was used to draw the network diagram of "component-target-pathway-function" (Shannon et al., 2003; Killcoyne et al., 2009).

**References**

Bairoch, A., Apweiler, R., Wu, C. H., Barker, W. C., Boeckmann, B., Ferro, S., Gasteiger, E., Huang, H., Lopez, R., Magrane, M., Martin, M. J., Natale, D. A., O'Donovan, C., Redaschi, N., Yeh, L. S. 2005. The Universal Protein Resource (UniProt). Nucleic Acids Research, 33(Database issue): D154-9. <https://doi:10.1093/nar/gki070.>

He, X., Wang, J., Li, M., Hao, D., Yang, Y., Zhang, C., He, R., Tao, R. 2014. Eucommia ulmoides Oliv.: ethnopharmacology, phytochemistry and pharmacology of an important traditional Chinese medicine. Journal of Ethnopharmacology, 151 (1): 78-92. <https://doi:10.1016/j.jep.2013.11.023.>

Kanehisa, M., Furumichi, M., Tanabe, M., Sato, Y., Morishima, K. 2017. KEGG: new perspectives on genomes, pathways, diseases and drugs. Nucleic Acids Research, 45 (D1): D353-D361. https://doi:10.1093/nar/gkw1092.

Killcoyne, S., Carter, G. W., Smith, J., Boyle, J. 2009. Cytoscape: a community-based framework for network modeling. Methods Mol Biol, 563: 219-239. https://doi:10.1007/978-1-60761-175-2_12.

Kim, S., Thiessen, P. A., Bolton, E. E., Chen, J., Fu, G., Gindulyte, A., Han, L., He, J., He, S., Shoemaker, B. A., Wang, J., Yu, B., Zhang, J., Bryant, S. H. 2016. PubChem Substance and Compound databases. Nucleic Acids Research, 44(D1): D1202-13. <https://doi:10.1093/nar/gkv951.>

Ru, J., Li, P., Wang, J., Zhou, W., Li, B., Huang, C., Li, P., Guo, Z., Tao, W., Yang, Y., Xu, X., Li, Y., Wang, Y., Yang, L. 2014. TCMSP: a database of systems pharmacology for drug discovery from herbal medicines. Journal of Cheminformatics, 6: 13. <https://doi:10.1186/1758-2946-6-13.>

Shannon, P., Markiel, A., Ozier, O., Baliga, N. S., Wang, J. T., Ramage, D., Amin, N., Schwikowski, B., Ideker, T. 2003. Cytoscape: a software environment for integrated models of biomolecular interaction networks. Genome Research, 13 (11): 2498-2504. https://doi:10.1101/gr.1239303.

Tao, W., Xu, X., Wang, X., Li, B., Wang, Y., Li, Y., Yang, L. 2013. Network pharmacology-based prediction of the active ingredients and potential targets of Chinese herbal Radix Curcumae formula for application to cardiovascular disease. Journal of Ethnopharmacology, 145(1): 1-10. <https://doi:10.1016/j.jep.2012.09.051.>

Wang, X., Shen, Y., Wang, S., Li, S., Zhang, W., Liu, X., Lai, L., Pei, J., Li, H. 2017. PharmMapper 2017 update: a web server for potential drug target identification with a comprehensive target pharmacophore database. Nucleic Acids Research, 45 (W1): W356-W360. https://doi:10.1093/nar/gkx374.

Wang, Y., Yu, W., Shi, C., Jiao, W., Li, J., Ge, J., Hong, Y., Shi, G. 2019. Network Pharmacology of Yougui Pill Combined with Buzhong Yiqi Decoction for the Treatment of Sexual Dysfunction. Evidence Based Complement Alternat Medicine, 2019: 1243743. https://doi:10.1155/2019/1243743.

Xu, X., Zhang, W., Huang, C., Li, Y., Yu, H., Wang, Y., Duan, J., Ling, Y. 2012. A novel chemometric method for the prediction of human oral bioavailability. International Journal of Molecular Sciences, 13(6): 6964-82. <https://doi:10.3390/ijms13066964.>

**Figure and table caption**

FIGURE S1. HPLC assay of CGA in EFEE. (A) The chromatogram of EFEE. (B-F) The chromatogram of CGA concentration from 2.04 to 408 μg/L. (G) The standard curve of CGA, and the content of CGA in EFEE.

TABLE S1. qRT-PCR Primers

TABLE S2. HPLC conditions
